# Respiratory failure presenting in H1N1 influenza with Legionnaires disease: two case reports

**DOI:** 10.1186/1752-1947-5-520

**Published:** 2011-10-21

**Authors:** Michele Iannuzzi, Edoardo De Robertis, Ornella Piazza, Fabio Rispoli, Giuseppe Servillo, Rosalba Tufano

**Affiliations:** 1 Dipartimento di Scienze Chirurgiche Anestesiologiche Rianimatorie e dell' Emergenza- Dipartimento di Anestesia e Rianimazione, Facoltà di Medicina e Chirurgia Federico II, Napoli, Italy

**Keywords:** Respiratory failure, viral infection, Legionella, swine flu

## Abstract

**Introduction:**

Media sensationalism on the H1N1 outbreak may have influenced decisional processes and clinical diagnosis.

**Case Presentation:**

We report two cases of patients who presented in 2009 with coexisting H1N1 virus and Legionella infections: a 69-year-old Caucasian man and a 71-year-old Caucasian woman. In our cases all the signs and symptoms, including vomiting, progressive respiratory disease leading to respiratory failure, refractory hypoxemia, leukopenia, lymphopenia, thrombocytopenia, and elevated levels of creatine kinase and hepatic aminotransferases, were consistent with critical illness due to 2009 H1N1 virus infection. Other infectious disorders may mimic H1N1 viral infection especially Legionnaires' disease. Because the swine flu H1N1 pandemic occurred in Autumn in Italy, Legionnaires disease was to be highly suspected since the peak incidence usually occurs in early fall. We do think that our immediate suspicion of Legionella infection based on clinical history and X-ray abnormalities was fundamental for a successful resolution.

**Conclusion:**

Our two case reports suggest that patients with H1N1 should be screened for Legionella, which is not currently common practice. This is particularly important since the signs and symptoms of both infections are similar.

## Introduction

Media sensationalism with respect to the swine flu outbreak may have influenced decisional processes and clinical diagnosis. We report two cases of patients who present during 2009 in whom H1N1 and Legionella infection coexisted. Secondary bacterial pneumonia is recognized as one of the most common causes of death in influenza cases. Coinfection has been found in 30% of all influenza cases in persons with seasonal influenza. The pathogens most often involved are *Streptococcus pneumoniae*, *Staphylococcus aureus*, and *Haemophilus influenza *[1,2]. From July 2009 through February 2010 in Italy, 2500 confirmed cases of pandemic influenza and four and a half million cases of influenza-like illnesses were reported to the sentinel surveillance system.

A total of 1278 (50%) confirmed cases of H1N1 were hospitalized. Of these, 271 (21%) cases presented with pneumonia, which was attributed to bacterial coinfection in 33 cases. Of the 33 cases with pneumonia due to a bacterial coinfection, six (18%) were due to the Legionella pneumophila serogroup 1 [3].

### Case 1

Our first case is a 69-year-old Caucasian man with a past medical history of coronary artery disease, chronic renal insufficiency, hypertension and type 1 diabetes. Two weeks earlier, he had been exposed to a child with an upper respiratory infection. He lived in a rural area. He had no history of insect bites, but was exposed to farm animals and pond water.

He had been well until nine days earlier, when dry cough, myalgias, fever (39.4°C), malaise, sore throat and nasal congestion presented. On physical examination in the Emergency Room (ER), the patient had a temperature of 39.2°C, a heart rate of 50 beats per minute and a respiratory rate of 35 breaths per minute. A buccal swab was negative for influenza A and B antigens, and no parasites were seen on a peripheral-blood smear. Acetaminophen, ketorolac, levofloxacin and normal saline were administered.

After 24 hours he presented with persistent fever (39.0°C), dry cough and respiratory failure and was admitted to the intensive care unit (ICU). Vital signs were as follows: blood pressure 135/70 mm Hg; heart rate 50 beats per minute; respiratory rate 34 breaths per minute; oxygen saturation 88%, on 50% inspired oxygen. On physical examination rhonchi were detected in the lower lung fields.

Repeated tests of nasopharyngeal secretions for influenza viruses, parainfluenza virus, respiratory syncytial virus, and adenovirus were negative. Testing for antibodies to toxoplasma was suggestive of past infection. Cultures of specimens of blood, urine, and sputum were sterile. Polymerase chain reaction determination of buccal swab for H1N1 influenza A virus was positive. Urinary tests for Legionella antigens were positive.

On admission main laboratory examations were as follows: white blood cell count (WBC) was 9.8 K/mL (87% neutrophils, 4% lymphocytes); C-reactive protein 205 mg/L; serum sodium 132 mEq/L; serum phosphorus 2.3 mg/dL; serum glutamate pyruvate transaminase (SGPT) 175 IU/L; serum oxaloacetate transaminase (SGOT) 184 IU/L; serum ferritin 4100 ng/mL; creatinine phosphokinase (CPK) 241 IU/L. Winthrop scale score was > 15.

**Figure 1:**
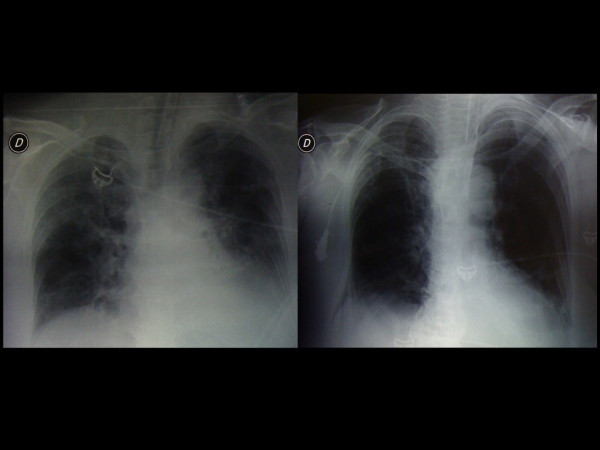
**Right panel: antero-posterior chest x-ray of patient 1**. Left panel: antero-posterior chest x-ray of patient 2.

Chest radiograph showed low lung volumes, with patchy air-space disease consistent with multifocal pneumonia (Figure 1). Intravenous azithromycin (500 mg twice daily), levofloxacin (500 mg twice daily) and oral oseltamivir (150 mg twice daily) were administered. Within 18 hours after arrival, tachypnea and hypoxemia (PaO_2_: 58 mm Hg, while breathing 50% oxygen) increased further requiring intubation and mechanical ventilation. Hypotension and renal failure developed; methylprednisolone and vasopressors were administered.

On the third day reverse transcriptasepolymerase chain reaction (RT-PCR) on a broncoalveolar lavage specimen was still positive for H1N1 influenza infection. Legionella antigens were also confirmed positive. On the fifth day he was extubated and non-invasive ventilation was started. He was discharged from the ICU on day 21.

### Case 2

Our second case is a 71-year-old Caucasian woman with a past medical history significant for hypertension, type 1 diabetes and chronic hepatitis C.

She reported an eight day history of dry cough and fever (39.2°C) associated with sore throat and nasal congestion. She lived in a peripheral urban area. On emergency room examination, her Glasgow Coma Score (GCS) was 12 (E = 3 V = 4 M = 5), temperature was 39.6°C, blood pressure was 80/40 mm Hg, heart rate was 54 beats per minute, respiratory rate was 40 breaths per minute and oxygen saturation was 88% on 50% inspired oxygen. Her chest radiograph was consistent with multifocal pneumonia (Figure 1). There were rhonchi in the left and right lung fields. A rapid test of a specimen from a buccal swab was negative for influenza A and B antigens, and no parasites were seen on a peripheral-blood smear. Acetaminophen, ketorolac, levofloxacin and normal saline were administered.

After one hour she was admitted to the ICU and due to worsening hypoxemia (PaO2 48 mm Hg, while breathing 50% inspired oxygen), neurological impairment with a GCS of 10 (E = 2 V = 3 M = 5) and hemodynamic instability (blood pressure 80/40 mmHg, heart rate 46 beats per minute), she was intubated and mechanically ventilated. RT-PCR on a broncoalveolar lavage specimen was positive for H1N1 influenza infection.

On admission main laboratory examination results were as follows: white blood cell (WBC) count was 5.8 K/Ml (77% neutrophils; 5% lymphocites); C-reactive protein was 312 mg/L; serum sodium was 129 mEq/L; serum phosphorus was 2.5 mg/dL; serum SGPT were 215 UI/L; serum SGOT were 220 UI/L; serum ferritin was 5280 ng/mL; CPK was 445 IU/L. Her Winthrop scale score was > 15.

Intravenous levofloxacin (500 mg twice daily) and oral oseltamivir (150 mg twice daily) were administered. On the second day, hypoxemia, hypotension and renal failure developed; norepinephrine was administered after fluid challenge. For persistent hypoxemia she was ventilated in the prone-supine position for 12 hour intervals daily. Tests of the urine for legionella antigens were positive. Azithromycin (500 mg twice daily) was added her treatment.

On the ninth day she underwent percutaneous tracheostomy. She was discharged from the ICU on day 35.

## Discussion

During the Spring of 2009, a novel influenza A (H1N1) virus of swine origin emerged to cause infections in humans in North America [4].

The pandemic was carefully followed by the media but with a touch of sensationalism that caused a widespread a sense of fear in the population. Health care systems and physicians were suddenly in the spotlight. In our cases all the signs and symptoms (including respiratory failure, refractory hypoxemia, leukopenia, lymphopenia, thrombocytopenia, and elevated levels of creatine kinase and hepatic aminotransferases) were consistent with critical illness due to infection with the 2009 H1N1 virus [1,4,5].

Other infectious disorders may mimic H1N1 viral infection especially Legionnaires' disease. Because the swine flu H1N1 pandemic occurred in Autumn in Italy, Legionnaires' disease was to be highly suspected since its peak incidence usually occurs in early fall.

Initial attempts to diagnose H1N1 infection using immunochromatography relied on test kits developed for seasonal influenza A and B viruses, many of which proved significantly less sensitive to H1N1. Hence, tests with monoclonal antibodies that react with H1N1 but not seasonal influenza A (H1N1 and H3N2) or B viruses were developed.

Recognizing viral hemagglutinin and nucleoprotein, specifically allows the detection of H1N1 virus in nasal wash fluid or nasopharyngeal fluid from patients with influenza-like illnesses.

Early and rapid diagnosis of H1N1-related respiratory insufficiency needs rapid screening during a pandemic but clinicians cannot rely only on the buccal swab test and need to rule out false positive and negative cases by RT-PCR on oral/nasal fluids or bronchoalveolar lavage specimens.

In case one the visit by a child with an upper respiratory infection five days before the onset of illness in the patient represents also a plausible exposure to the 2009 H1N1 virus; in case two the lack of positive anamnesis for other suspicions together with the finding of positive specimens for H1N1 influenza A infection could have caused us arrive at a fashionable diagnosis and stopped us from further investigations. We do think that the immediate suspicion of Legionella infection based on clinical history, X-ray abnormalities and Winthrop University Hospital Infectious Disease Division's diagnostic weighted point system scale (Table 1) were fundamental for a successful resolution [6,7].

**Table 1:** Winthrop-University Hospital Infectious Disease Division's diagnostic weighted point system for diagnosing Legionnaires' disease in adults (modified [15])

Presentation	Point score
**Clinical features**	

Temperature > 102 F* With relative bradycardia	5

Headache Active onset	2

Mental confusion/lethargy* Not drug-induced	4

Ear pain Acute onset	3

Nonexudative pharyngitis Acute onset	3

Hoarseness Acute not chronic	3

Sputum (purulent) Excluding AECB	3

Hemoptysis* Mild/moderate	3

Chest pain (pleuritic)	3

Loose stools/watery diarrhea* Not drug-induced	3

Abdominal pain* With/without diarrhea	2

Renal failure* Acute (not chronic)	3

Shock/hypotension* Excluding cardiac/pulmonary causes	5

Splenomegaly Excluding non-CAP causes	5

Lack of response to B-lactam antibiotics after 72 hours (excluding viral pneumonias)	5

Laboratory tests	

Chest x-ray Rapidly progressive asymmetric infiltrates* (excluding severe influenza (human, avian, swine), HPS, SARS)	3

Severe hypoxemia with (A-a gradient (> 35)* Acute onset	2

Hyponatremia* Acute onset	1

Hypophosphatemia Acute onset	5

Elevated SGOT/SGPT (early/mild/transient)* Acute onset	2

Elevated Total bilirubin Acute onset	1

Elevated LDH (> 400)* Acute onset	5

Elevated CPK* Acute onset	4

Elevated CRP* Acute onset	5

Elevated Cold agglutinin titers (1:64) Acute onset	5

Relative lymphopenia (< 21%)* Acute onset	5

Elevated Ferritin (> 2 n)*	5

Microscopic hematuria* Excluding trauma, BPH, Foley catheter, bladder and/or renal neoplasms	2

Total point score Legionnaires' Disease very likely > 15 Legionnaires' Disease likely 5-15 Legionnaires' Disease unlikely < 5	

AECB, acute exacerbation of chronic bronchitis; BPH, benign prostatic hyperplasia; CPK, creatine phosphokinase; CRP, C-reactive protein; HPS, Hantavirus pulmonary syndrome; LDH, lactate dehydrogenase; SARS, severe acute respiratory syndrome; SGOT/SGPT, serum glutamate oxaloacetate transaminase/serum glutamate pyruvate transaminase; *otherwise unexplained (acute and associated with the pneumonia).

Almost all patients affected by pandemic H1N1 infections admitted to an ICU because of lung involvement receive empiric antibiotic therapy. However, preliminary clinical data have failed to demonstrate a consistent role of bacterial co-infection suggesting that severe pulmonary damage occurs as a result of viral pneumonia [1,11]. A recent autopsy study revealed evidence of concurrent bacterial infection in 29% of cases [8]. In 45% of these the pathogen was *S. pneumoniae*. These findings confirm the results of previous studies of autopsy specimens showing that most deaths attributed to influenza A virus occurred concurrently with bacterial pneumonia [9]. On the other hand, they highlight the importance of treating influenza patients with both empiric antibacterial therapy and antiviral medications. According to our experience we believe that zoonotic infections had to be ruled out. The lack of known contact with animals in an immunocompetent host appears to rule out zoonotic infections, such as *Coxiella burnetii*. One of the patients had worked near water ponds, which could have been contaminated by animal urine, but he did not have pulmonary hemorrhage so leptospirosis seems unlikely. Without exposure to birds, *Chlamydia psittaci *is unlikely. Community-acquired pneumonia (*Streptococcus pneumoniae, Haemophilus influenzae, S. pyogenes, or Staphylococcus aureus*) can cause severe pulmonary disease, especially in patients with antecedent influenza. These pathogens should have responded to the broad-spectrum antimicrobial therapy; therefore, they are unlikely to have been the sole cause of illness. Atypical bacterial pathogens such as *Legionella pneumophila *cause multifocal pneumonia but usually do not cause upper respiratory tract symptoms [1,2].

Anaplasmosis could result in a lower respiratory tract disease but fulminant disease is rare and clinical improvement should have occurred with levofloxacin treatment [10]. Radiographic abnormalities also needed to be ruled out. Many critically ill patients have radiographic findings of viral pneumonitis, with bilateral interstitial and alveolar infiltrates. Multifocal and patchy abnormalities as seen in these patients have been reported in cases of 2009 H1N1 influenza A virus infection but do not completely rule out invasive bacterial infection [2]. Ground glass opacity and cavitary lobar opacity should focus attention on Legionnaire's disease [11].

Another potential contributory factor that needed to be ruled out was viral infection of the respiratory tract. Infection with adenovirus or influenza virus must be considered. Adenovirus type 14 is the most likely cause of severe viral pneumonia in adults. Radiographic findings may include lobar infiltrates, although these are more characteristic of bacterial pneumonia [12,13]. The fact that bacterial infections should have responded to levofloxacin argues against the fact that a secondary bacterial pneumonia superimposed with influenza A or B causing severe pulmonary disease. The lack of recent travel in H5N1 (bird flu) endemic areas or exposure to sick or dead poultry argue against H5N1 influenza (bird flu) [14].

## Conclusions

Our two case reports suggest that patients with H1N1 should be screened for Legionella, which is not currently common practice. This is particularly important since the signs and symptoms of both infections are similar. Doctors should never be dazzled by contingency and media sensationalism in decision making. With prompt identification of the bacterial etiology of pneumonia, appropriate treatment can be started with both antibacterial therapy and antiviral medications. The length of hospital stay and the mortality of both pandemic and seasonal influenza can be reduced.

## Consent

Written informed consent was obtained from both patients for publication of this case report and any ccompanying images. A copy of the written consent is available for review by the Editor-in-Chief of this journal.

## Competing interests

The authors declare that they have no competing interests.

## Authors' contributions

MI collected patient data regarding the ER and ICU and was a major contributor in writing the manuscript. OP was responsible for buccal swab and bronchoalveolar lavage specimens and for RT-PCR processing. FR interpreted radiological findings and provided the radiological differential diagnosis. GS provided a major contribution in data analysis and interpretation. RT provided a major contribution in data analysis and interpretation.

EDR collected data regarding ER and ICU, contributed to the analysis and interpretation, and was a major contributor in writing the manuscript. All authors read and approved the final manuscript.
